# Approach to Detecting Beneficial and Detrimental Drug-Drug Interactions in Complex Pharmacotherapy

**DOI:** 10.7759/cureus.78746

**Published:** 2025-02-08

**Authors:** Toru Ogura, Chihiro Shiraishi

**Affiliations:** 1 Clinical Research Support Center, Mie University Hospital, Tsu, JPN; 2 Pharmacy, Mie University Hospital, Tsu, JPN

**Keywords:** adverse event, database, medication management, patient safety, polypharmacy, statistical modeling

## Abstract

Background

The increasing prevalence of polypharmacy has raised concerns about drug-drug interactions (DDIs) and their impact on patient safety. Database-based DDI detection often suffers from insufficient patient background information and missing data, limiting the accuracy and applicability of DDI assessments. A novel model is needed to overcome these limitations and provide a more comprehensive evaluation of DDIs to enhance patient safety in the context of multiple medication use.

Objectives

This study aims to develop and validate a novel model for evaluating both the beneficial and detrimental effects of DDIs on patient safety. The model is designed to address challenges associated with insufficient patient background information and missing data in database studies while providing a comprehensive assessment of DDIs using statistical inference and hypothesis tests.

Methods

To address the challenges of insufficient patient background information and missing data often encountered in database studies, the proposed model incorporates an overlap parameter. This parameter represents the degree of commonality in patient profiles susceptible to adverse events from individual drug administrations. The magnitude of DDIs is presented in a 2×2 contingency table constructed by the occurrence or non-occurrence of specific adverse events in observed value and expected value estimated from the model. This tabular format facilitates the assessment of DDIs using statistical inference and hypothesis tests.

Results

Simulations under various settings confirmed that significance levels for statistical hypothesis tests were strictly observed. Furthermore, applications to real-world databases demonstrated that the proposed model effectively identifies both positive and negative DDIs.

Conclusions

This research provides healthcare professionals with a robust and practical tool for enhanced DDI detection and management. The presentation of findings in a familiar 2×2 contingency table format improves the accessibility of our results, facilitating straightforward interpretation. The proposed model has the potential to promote a safer healthcare environment for patients on multiple medications, ultimately enhancing patient safety and treatment efficacy.

## Introduction

Drug-drug interactions (DDIs) represent a significant challenge in modern medicine, particularly as polypharmacy becomes increasingly prevalent, especially among older adults [1,2]. The identification and assessment of these DDIs are crucial for safeguarding patient well-being and maximizing therapeutic efficacy. This importance is underscored by instances where DDIs have resulted in severe adverse events [3, 4]. While clinical trials often designate potentially interacting drugs as prohibited concomitant medications [5], this approach does not provide evidence of actual DDIs. In real-world clinical settings, patients frequently receive various drug combinations, prompting researchers to increasingly utilize large-scale databases to investigate potential DDIs and their clinical implications [5,6].

For the purpose of DDI detection, this study defines four treatment groups: the "drug A group" (administration of drug A alone), the "drug B group" (administration of drug B alone), the "drugs AB group" (co-administration of drugs A and B), and the "no AB group" (administration of neither drug A nor drug B). The sample sizes for these groups are denoted as n_A _≥ 1, n_B _≥ 1, n_AB _≥ 1, and n_no AB _≥ 1, respectively. Within each group, the number of patients experiencing a specific adverse event is represented by x_A_ (0 ≤ x_A _≤ n_A_), x_B_ (0 ≤ x_B _≤ n_B_), x_AB_ (0 ≤ x_AB _≤ n_AB_), and x_no AB_ (0 ≤ x_no AB _≤ n_no AB_). The observed proportion of patients experiencing the specific adverse event in each group is calculated as q_A _= x_A _/ n_A_, q_B _= x_B _/ n_B_, q_AB _= x_AB _/ n_AB_, and q_no AB _= x_no AB _/ n_no AB_. We denote the expected proportion derived from a model as r_AB_. Furthermore, we define the population proportions for drug A, drug B, and drugs AB groups as p_A_, p_B_, and p_AB_, respectively. To ensure clarity and prevent confusion, we employ distinct notations for observed, expected, and population proportions. We assume that q_A _≤ q_B_, without loss of generality. This assumption is justified as we can always interchange the notations of drugs A and B if q_A _> q_B_ occurs, thereby satisfying the condition q_A _≤ q_B_.

Various models have been proposed for DDI signal detection in large-scale pharmacovigilance databases, including additive, multiplicative, and logistic mixed-effect models [5,7]. The additive model assumes that the effects of two drugs are independent and additive. The expected proportion in the drugs AB group can be calculated as r_AB _= q_A _+ q_B _- q_no AB_. This equation represents the sum of the individual drug effects (q_A_ and q_B_) minus the baseline risk (q_no AB_) to avoid double counting the background risk. The multiplicative model assumes that the relative risks are multiplicative. The expected proportion in the drugs AB group can be expressed as r_AB _= 1 - (1 - q_A_)(1 - q_B_) / (1 - q_no AB_). This formula calculates the proportion of experiencing the specific adverse event when both drugs are administered, assuming their effects multiply rather than add. A representative example of a logistic mixed-effect model [5] using a database can be expressed by the following equation as log(r_AB _/ (1 - r_AB_)) = β_0 _+ β_1_x_A _+ β_2_x_B _+ β_3_x_A_:x_B _+ β_4_z_1 _+ β_5_z_2_, where x_A_:x_B_ denotes the interaction term between variables x_A_ and x_B_, z_1_ represents sex, and z_2_ represents age. The coefficients of β_0_, β_1_, β_2_, β_3_, β_4_, and β_5_ correspond to the intercept, x_A_, x_B_, x_A_:x_B_, z_1_, and z_2_, respectively. However, these existing models have several limitations. In additive and multiplicative models, a primary concern stems from the indiscriminate inclusion of patients in the no AB group, potentially leading to significant biases in baseline risk estimation. This heterogeneous inclusion could artificially alter the value of q_no AB_, potentially distorting both additive and multiplicative models and consequently affecting the assessment of DDIs. While the incorporation of diverse real-world data may enhance model applicability, it raises concerns about comparability and might introduce unnecessary complexity and potentially create rather than mitigate confounding effects. In logistic mixed-effect models, a significant limitation often stems from the absence of comprehensive patient information, such as detailed medical histories, in databases [8]. This frequently results in a paucity of patient background variables that can be incorporated into the model. Even for fundamental patient background variables such as sex and age, the prevalence of missing data can range from 5% to 20% in large-scale databases [9]. Consequently, the logistic mixed-effect models may not function optimally.

This study aims to develop a novel model applicable to database studies that evaluates both the beneficial and detrimental effects of DDIs on patient safety. To address issues of existing models, the proposed model incorporates an overlap parameter that represents the degree of commonality in patient profiles susceptible to adverse events from individual drug administrations. By presenting results in a 2×2 contingency table format, this approach facilitates the application of various statistical methods across different types of adverse event databases. The effectiveness of the proposed model will be validated through simulations and applications to real-world databases, ensuring its applicability across diverse clinical settings. Ultimately, this research seeks to contribute to improved DDI detection and management, thereby enhancing patient safety and treatment efficacy.

## Materials and methods

Novel model

**Figure 1:**
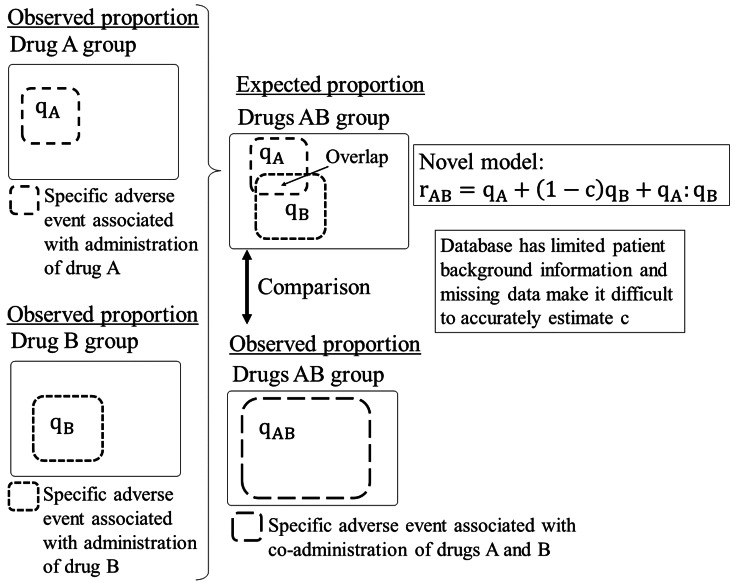
Process of deriving a novel model c: overlap parameter; q_A_: observed proportion of patients with the specific adverse event in the drug A group; q_B_: observed proportion of patients with the specific adverse event in the drug B group; q_AB_: observed proportion of patients with the specific adverse event in the drug AB group; q_A_:q_B_: sample-level interaction term between drugs A and B; r_AB_: expected proportion of patients experiencing the specific adverse event in the drugs AB group, derived from a model.

**Figure 2:**
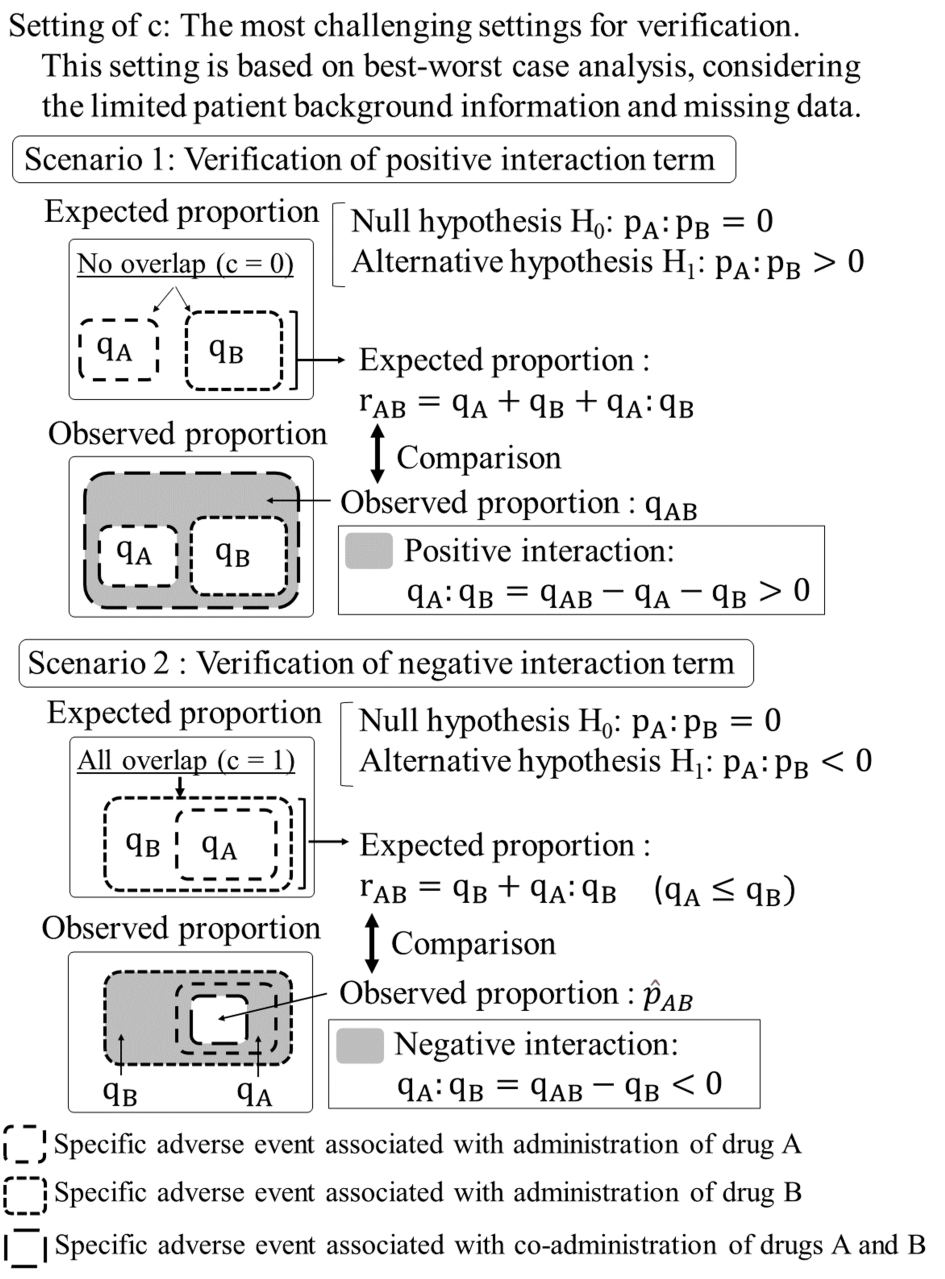
Verification of interaction term in Scenarios 1 and 2 c: overlap parameter; p_A_:p_B_: population-level interaction term between drugs A and B; q_A_: observed proportion of patients with the specific adverse event in the drug A group; q_B_: observed proportion of patients with the specific adverse event in the drug B group; q_AB_: observed proportion of patients with the specific adverse event in the drug AB group; q_A_:q_B_: sample-level interaction term between drugs A and B; r_AB_: expected proportion of patients experiencing the specific adverse event in the drugs AB group, derived from a model.

We propose a novel model for detecting DDI, the derivation process of which is illustrated in Figures 1, 2. Unlike existing multivariate analysis models that often face limitations due to insufficient covariates, the proposed model introduces an overlap parameter c (0 ≤ c ≤ 1) as a proxy for covariates. This parameter quantifies the degree of commonality in patient profiles susceptible to specific adverse events from individual drug administrations. The model is mathematically expressed as rAB = qA + (1 - c)qB + qA:qB, where qA:qB denotes the interaction term between qA and qB. In database studies, limited patient background information and missing data present significant challenges in accurately estimating the overlap parameter c. To address this issue, we determine the c value based on the best-worst-case analysis of missing data [10]. This approach corresponds to using c = 0 for verifying the positive interaction term (Scenario 1) and c = 1 for verifying the negative interaction term (Scenario 2), thereby establishing the most challenging conditions conceivable for verification. If either the positive or negative interaction terms can be validated under these stringent conditions, the results can be considered highly reliable.

The selection of the appropriate scenario is determined by the observed proportions of the specific adverse events for drug A, drug B, and drugs AB groups. When q_A_ + q_B_ < q_AB_, we investigate a potential positive DDI using Scenario 1 (c = 0). Conversely, when q_AB_ < q_B_, we investigate a potential negative DDI using Scenario 2 (c = 1). In instances where q_B_ ≤ q_AB_ ≤ q_A_ + q_B_, neither scenario is applicable, and DDI verification is not pursued. It is imperative to note that the inability to verify a DDI in the last case does not necessarily imply its absence. This situation indicates that the proposed model, under the given constraints, cannot conclusively demonstrate the presence of a DDI. This approach ensures that we investigate DDIs only when the observed data suggests their potential existence, thereby focusing our analysis on the most promising cases. This scenario selection process aligns with our best-worst-case analysis strategy, allowing us to examine DDIs under the most challenging conditions when the data suggests their potential existence. We maintain a conservative approach to DDI detection, prioritizing the reliability of our findings in the context of limited covariate information.

In Scenario 1, we assume no overlap in patient profiles associated with the specific adverse event between the two drugs (c = 0). For instance, the specific adverse event in the drug A group occurs only in males, while the same adverse event in the drug B group occurs only in females. In this case, it is expected that this specific adverse event will occur in both males and females in the drugs AB group. The expected proportion in the drugs AB group is r_AB _= q_A _+ q_B _+ q_A_:q_B_.

In Scenario 2, we assume complete overlap in patient profiles associated with the specific adverse event between the two drugs (c = 1). For instance, the specific adverse event occurs only in elderly men within both drug A and drug B groups, with no specific adverse event reported in patients from other categories. In this case, it is expected that this specific adverse event in the drug AB group will manifest only in elderly men. While we have illustrated the overlap using sex and age as examples, this assumption posits that all patient profiles contribute to the specific adverse event even if the information is not explicitly provided in the database. The expected proportion in the drugs AB group is r_AB _= q_B _+ q_A_:q_B_ (note that we assume q_A _≤ q​_B​​​​​_).

This approach facilitates a comprehensive assessment of potential DDIs while addressing the inherent constraints of database studies, notably the lack of specific covariates that could impact outcomes. By calibrating the overlap parameter to establish stringent criteria for validating the interaction term, we can derive robust conclusions. This framework offers a rigorous method for evaluating DDIs, even when faced with limited covariate data, thereby enhancing the credibility of our findings within the context of database-driven research.

Furthermore, observed and expected values can be displayed in 2×2 contingency tables (Tables 1, 2 for Scenarios 1 and 2, respectively), facilitating the calculation of various statistical quantities. Databases can be broadly categorized into two types: those that do not include data on patients without adverse events, such as the United States Food and Drug Administration Adverse Event Reporting System (FAERS) [11], and those that do include such data, like Sentinel Initiative [12]. For databases like FAERS, methods such as reporting odds ratio (ROR) [13,14], reporting risk ratio [15], and proportional reporting ratio [16] can be readily applied. For databases like Sentinel Initiative, methods such as odds ratio [17], relative risk [17], Pearson's chi-squared test, and Fisher's exact test can be utilized. The ability to apply such a wide range of statistical techniques enhances the model's utility across diverse pharmacovigilance datasets, thereby increasing its practical value in real-world drug safety assessments.

**Table 1:** 2×2 contingency table in Scenario 1 (c = 0) x_AB_: number of patients experiencing the specific adverse event in the drugs AB group; n_AB_: sample size of the drugs AB group; q_A_: observed proportion of patients with the specific adverse event in the drug A group; q_B_: observed proportion of patients with the specific adverse event in the drug B group.

	Specific adverse event	No specific adverse event
Observed value	x_AB_	n_AB _- x_AB_
Expected value	n_AB_(q_A _+ q_B_)	n_AB _- n_AB_(q_A _+ q_B_)

**Table 2:** 2×2 contingency table in Scenario 2 (c = 1) x_AB_: number of patients experiencing the specific adverse event in the drugs AB group; n_AB_: sample size of the drugs AB group; q_B_: observed proportion of patients with the specific adverse event in the drug B group.

	Specific adverse event	No specific adverse event
Observed value	x_AB_	n_AB _- x_AB_
Expected value	n_AB_q_B_	n_AB _- n_AB_q_B_

Statistical hypothesis test

Due to the different c values used in testing p_A_:p_B _> 0 and testing p_A_:p_B_ < 0, the statistical hypothesis test was constructed as two one-sided tests with a significance level of 0.025 each, maintaining an overall significance level of 0.05. In this context, p_A_:p_B_ represents the interaction term between p_A_ and p_B_ in the population. This approach allows for a more comprehensive evaluation of DDIs while accounting for limitations in available data and providing a framework for assessing both positive and negative interaction terms.

The statistical hypothesis test in Scenario 1 aims to verify a positive interaction term. The null hypothesis is denoted as H_0_: p_A_:p_B_ = 0,​ while the alternative hypothesis is expressed as H_1_: p_A_:p_B _> 0, employing a one-sided test with a significance level of 0.025. The evaluation of the statistical hypothesis test is based on the p-value, which represents the probability of observing the obtained results, or more extreme outcomes, under the assumption that the null hypothesis is true. This test framework is rigorously adhered to in our approach. Consequently, the expected values are calculated under the condition p_A_:p_B_ = 0. Table 1 presents a 2×2 contingency table displaying the occurrence or non-occurrence of specific adverse events in terms of observed and expected values. When the expectation value is set to reference, the ROR of the observed value is calculated as ROR = [x_AB _/ (n_AB _- x_AB_)] / {n_AB_(q_A _+ q_B_) / [n_AB _- n_AB_(q_A _+ q_B_)]}. Based on a one-sided significance level of 0.025, the lower bound of the 97.5% confidence interval (CI) was calculated as exp(log(ROR) - 1.96{1 / x_AB_+1 / (n_AB _- x_AB_) + 1 / [n_AB_(q_A _+ q_B_)] + 1 / [n_AB _- n_AB_(q_A _+ q_B_)]}^1/2^). The p-value of ROR is calculated as 1 - f(log(ROR) / {1 / x_AB _+ 1 / (n_AB _- x_AB_) + 1 / [n_AB_(q_A _+ q_B_)] + 1 / [n_AB _- n_AB_(q_A _+ q_B_)]}^1/2^), where f(·) denotes the cumulative distribution function of standard normal distribution.

The statistical hypothesis test in Scenario 2 aims to verify a negative interaction term. The null hypothesis is denoted as H_0_: p_A_:p_B_ = 0, while the alternative hypothesis is expressed as H_1_: p_A_:p_B_ < 0​​​​​​, employing a one-sided test with a significance level of 0.025. Table 2 presents a 2×2 contingency table displaying the occurrence or non-occurrence of specific adverse events in terms of observed and expected values. Utilizing this table, various statistical hypothesis tests can be conducted to assess the significance of the interaction term. When the expectation value is set to reference, the ROR of the observed value is calculated as ROR = [x_AB_ / (n_AB_ - x_AB_)] / [n_AB_q_B_ / (n_AB_ - n_AB_q_B_)]. Based on a one-sided significance level of 0.025, the upper bound of the 97.5% CI was calculated as exp{log(ROR) - 1.96[1 / x_AB _+ 1 / (n_AB _- x_AB_) + 1 / n_AB_q_B _+ 1 / (n_AB _- n_AB_q_B_)]^1/2^}. The p-value of ROR is calculated as f{log(ROR) / {1 / x_AB _+ 1 / (n_AB _- x_AB_) + 1 / n_AB_q_B _+ 1 / (n_AB _- n_AB_q_B_)]^1/2^}​​​​​​. Other statistical hypothesis tests that can be performed from the 2×2 contingency table (such as Fisher's exact test) are also possible.

Simulation settings

The performance of the proposed model was evaluated through simulations. The efficacy of the model was evaluated based on adherence to a one-sided significance level of 0.025 for the Type I error rate and the magnitude of statistical power. These simulations aimed to assess the model's efficacy under circumstances that closely resemble the examples presented later in the Applications section. In Scenario 1, simulations were conducted with sample sizes n_A _= 10,000, n_B _= 5,000, and n_AB _= 1,000, with 10,000 iterations for each setting. Combinations of proportions (p_A_, p_B_) were selected ranging from 0.01 to 0.09. In Scenario 2, simulations were conducted with sample sizes n_A _= 10,000, n_B _= 5,000, and n_AB _= 2,500, with 10,000 iterations for each setting. Combinations of proportions (p_A_, p_B_) were selected ranging from 0.02 to 0.04. The Type I error rate was investigated under the null hypothesis (H_0_: p_A_:p_B_ = 0), while statistical power was examined under the alternative hypothesis (H_1_: p_A_:p_B_​​​​​​ > 0​​​​​​ for Scenario 1, and H_1_: p_A_:p_B_​​​​​​​ < 0​​​​​​​​​​​​​ for Scenario 2). In both scenarios, the test of ROR and Fisher’s exact test were performed as one-sided tests. The number of null hypothesis rejections (p ≤ 0.025) was counted for each test. The Type I error rate was calculated as the proportion of rejections out of total simulations, concluding that the nominal significance level was maintained when this Type I error was ≤ 0.025. Statistical power was similarly calculated for various interaction term values: p_A_:p_B_​ ​​​​​​= 0.01, 0.03, 0.05 for positive interaction terms, and p_A_:p_B_​ ​​​​​​​​​​​​​= -0.005, -0.010, -0.015 for negative interaction terms.

Application settings

The proposed model was applied to detect DDIs using FAERS data between the first quarter of 2004 (2004Q1) and 2024Q1. The FAERS data files were obtained from the official website on May 4, 2024. As Fisher's exact test is generally not considered appropriate for FAERS data, only the test of ROR was conducted in Applications 1 and 2. Application 1 examined the DDI between tramadol (drug A) and ciprofloxacin (drug B), focusing on three adverse events: acute kidney injury, renal failure, and chronic kidney disease. For each event, the ROR and its lower bound of the 97.5% CI were calculated. The test of ROR was employed to assess the statistical significance of potential positive interaction terms. Application 2 examined the DDI between furosemide (drug A) and perindopril (drug B), focusing on three adverse events: nausea, headache, and back pain. For each event, the ROR and its upper bound of the 97.5% CI were calculated. The test of ROR was employed to assess the statistical significance of potential negative interaction terms. Approval from an institutional review board was not required because the FAERS is an unlinkable, anonymized database that is open to the public.

Statistical software

The software R version 4.4.1 (R Foundation for Statistical Computing, Vienna, Austria) was used for all simulations and applications.

## Results

Simulation results

For Scenario 1, Tables 3, 4 summarize the Type I error rate and statistical power, respectively. Similarly, Tables 5, 6 summarize these metrics for Scenario 2. In both scenarios, we observed that the Type I error rates for both the test of ROR and Fisher’s exact test consistently remained ≤ 0.025 across all simulation settings. In terms of statistical power in Scenario 1, when p_A_:p_B_ = 0.01, power was consistently low across all simulation settings. For p_A_:p_B_ = 0.03, high power was observed when both p_A_ and p_B_ were low; however, power diminished as p_A_ and p_B_ increased. When p_A_:p_B_ = 0.05, high statistical power was maintained across all simulation settings. In terms of statistical power in Scenario 2, when p_A_:p_B_= -0.005, power was consistently low across all simulation settings. For p_A_:p_B_ = -0.010, high power was observed when both p_A_ and p_B_​ were low; however, the power diminished as p_A_ and p_B_ increased. When p_A_:p_B_ = -0.015, high statistical power was maintained across all simulation settings.

**Table 3:** Type I error rate for interaction term in Scenario 1 p_A_: population proportion of patients with the specific adverse event in the drug A group; p_B_: population proportion of patients with the specific adverse event in the drug B group; ROR: reporting odds ratio

p_A_	p_B_	ROR	Fisher
0.01	0.01	0.0022	0.0016
0.01	0.03	0.0030	0.0019
0.01	0.05	0.0030	0.0021
0.01	0.07	0.0049	0.0039
0.01	0.09	0.0050	0.0042
0.03	0.03	0.0025	0.0021
0.03	0.05	0.0053	0.0041
0.03	0.07	0.0052	0.0041
0.03	0.09	0.0045	0.0039
0.05	0.05	0.0040	0.0030
0.05	0.07	0.0043	0.0037
0.05	0.09	0.0058	0.0047
0.07	0.07	0.0046	0.0035
0.07	0.09	0.0049	0.0041
0.09	0.09	0.0040	0.0036

**Table 4:** Statistical power for interaction term in Scenario 1 pA: population proportion of patients with the specific adverse event in the drug A group; pB: population proportion of patients with the specific adverse event in the drug B group; ROR: reporting odds ratio

p_A_	p_B_	ROR			Fisher		
		0.01	0.03	0.05	0.01	0.03	0.05
0.01	0.01	0.2303	0.9881	1.0000	0.1816	0.9812	1.0000
0.01	0.03	0.1186	0.9022	0.9994	0.0947	0.8799	0.9992
0.01	0.05	0.0781	0.7704	0.9959	0.0631	0.7375	0.9939
0.01	0.07	0.0637	0.6609	0.9831	0.0535	0.6272	0.9801
0.01	0.09	0.0547	0.5777	0.9647	0.0450	0.5445	0.9597
0.03	0.03	0.0736	0.7814	0.9960	0.0612	0.7474	0.9946
0.03	0.05	0.0556	0.6616	0.9854	0.0456	0.6304	0.9813
0.03	0.07	0.0527	0.5704	0.9667	0.0423	0.5388	0.9602
0.03	0.09	0.0481	0.4877	0.9371	0.0400	0.4604	0.9280
0.05	0.05	0.0471	0.5755	0.9680	0.0385	0.5414	0.9618
0.05	0.07	0.0442	0.4887	0.9467	0.0361	0.4573	0.9384
0.05	0.09	0.0385	0.4433	0.9160	0.0322	0.4156	0.9039
0.07	0.07	0.0379	0.4430	0.9133	0.0327	0.4155	0.9040
0.07	0.09	0.0382	0.4009	0.8803	0.0330	0.3708	0.8664
0.09	0.09	0.0332	0.3628	0.8532	0.0290	0.3362	0.8373

**Table 5:** Type I error rate for interaction term in Scenario 2 p_A_: population proportion of patients with the specific adverse event in the drug A group; p_B_: population proportion of patients with the specific adverse event in the drug B group; ROR: reporting odds ratio

p_A_	p_B_	ROR	Fisher
0.02	0.02	0.0136	0.0113
0.02	0.03	0.0127	0.0103
0.02	0.04	0.0122	0.0101
0.03	0.03	0.0130	0.0107
0.03	0.04	0.0129	0.0112
0.04	0.04	0.0102	0.0087

**Table 6:** Statistical power for interaction term in Scenario 2 p_A_: population proportion of patients with the specific adverse event in the drug A group; p_B_: population proportion of patients with the specific adverse event in the drug B group; ROR: reporting odds ratio

p_A_	p_B_	ROR			Fisher		
		-0.005	-0.01	-0.015	-0.005	-0.01	-0.015
0.02	0.02	0.2435	0.8795	0.9999	0.2129	0.8506	0.9998
0.02	0.03	0.1605	0.644	0.9741	0.1419	0.6121	0.9687
0.02	0.04	0.1178	0.4857	0.8941	0.1029	0.4522	0.8785
0.03	0.03	0.1563	0.6525	0.9749	0.1354	0.6225	0.9707
0.03	0.04	0.1125	0.4785	0.8901	0.0999	0.4461	0.8733
0.04	0.04	0.1192	0.4807	0.8868	0.1035	0.4505	0.8684

 In both scenarios, the significance level was respected for both tests; however, power was low in certain instances. It is important to distinguish between statistical significance and clinical significance since statistically significant small interaction terms may not necessarily translate into clinically meaningful differences [18]. Consequently, cases identified as having low statistical power should not be automatically considered shortcomings of the method; rather, this characteristic may mitigate a potential drawback of detecting statistically significant yet clinically irrelevant differences. Conversely, for interaction terms of +0.05 and -0.015, which are more likely to be clinically significant, the high detection power suggests that the proposed model's test aligns statistical significance more closely with clinical significance. This alignment potentially enhances the test's practical utility in clinical settings, making it more user-friendly for healthcare professionals. The simulation results thus demonstrate the model's ability to maintain appropriate Type I error rates while providing meaningful statistical power for detecting clinically relevant DDIs.

Application 1 results

This study investigated the DDIs between tramadol (drug A) [19] and ciprofloxacin (drug B) [20] with respect to acute kidney injury, renal failure, and chronic kidney disease. Table 7 presents a summary of patient background variables (sex, age, and weight) for each group. The data exhibited considerable missing information, with 7% to 10% of sex data, 25% to 40% of age data, and 50% to 70% of weight data being absent. Given the significant amount of missing data in patient background variables, it was anticipated that estimating the c value from these variables or using them as covariates would not yield high-performance results. Table 7 also provides an overview of the proportions of adverse events by group. For acute kidney injury, the observed proportions were q_A _= 2.3%, q_B _= 3.8%, and q_AB _= 17.3%. For renal failure, the observed proportions were q_A _= 1.6%, q_B _= 2.7%, and q_AB _= 15.4%. For chronic kidney disease, the observed proportions were q_A _= 1.9%, q_B _= 2.9%, and q_AB _= 27.3%. To select the appropriate scenario, we checked whether q_A_ + q_B_ < q_AB_ was satisfied for these three adverse events. For acute kidney injury, q_A_ + q_B_ = 2.3% + 3.8% = 6.1% < q_AB_ = 17.3% Similarly, for renal failure, q_A_ + q_B_ = 1.6% + 2.7% = 4.3% < q_AB_ = 15.4%, and for chronic kidney disease, q_A_ + q_B_ = 1.9% + 2.9% = 4.8% < q_AB_ = 27.3%. As the condition was met for all three adverse events, they were considered potential candidates for positive DDIs. Consequently, the proposed model with c = 0 was employed to conduct two tests, and the results were summarized in Table 8. The ROR with the lower bound of the 97.5% CI and p-value for acute kidney injury were 3.198 (97.5% CI: 2.808, ∞), p < 0.001; for renal failure, they were 4.044 (97.5% CI: 3.484, ∞), p < 0.001; and for chronic kidney disease, they were 7.467 (97.5%: 6.512, ∞), p < 0.001. Positive DDIs were confirmed for these three adverse events.

**Table 7:** Summary of patient background variables for tramadol (drug A) and ciprofloxacin (drug B) Sex is summarized as frequency (proportion), age and weight are summarized as median (Q1–Q3), and missing data for each patient background variable are summarized as frequency (proportion). Q1: first quartile; Q3: third quartile

	Drug A group	Drug B group	Drugs AB group
	N = 204,368	N = 81,423	N = 5,535
Sex			
Female, n (%)	116,532 (57.0)	42,330 (52.0)	3,366 (60.8)
Male, n (%)	72,470 (35.5)	31,995 (39.3)	1,665 (30.1)
Missing, n (%)	15,366 (7.5)	7,098 (8.7)	504 (9.1)
Age (years)			
Median	60.0	59.0	62.0
Q1–Q3	47.0–71.0	43.0–71.0	51.0–69.0
Missing, n (%)	74,755 (36.6)	20,904 (25.7)	1,483 (26.8)
Weight, kg			
Median	76.0	73.0	79.4
Q1–Q3	63.0–91.2	60.0–87.2	64.8–95.0
Missing, n (%)	137,583 (67.3)	51,432 (63.2)	3,062 (55.3)
Adverse event			
Acute kidney injury, n (%)	4,685 (2.3)	3,131 (3.8)	958 (17.3)
Renal failure, n (%)	3,370 (1.6)	2,158 (2.7)	851 (15.4)
Chronic kidney disease, n (%)	3,838 (1.9)	2,371 (2.9)	1,511 (27.3)

**Table 8:** ROR and p-value for positive interaction term in each adverse event with tramadol (drug A) and ciprofloxacin (drug B) 97.5% CI: the lower bound of the 97.5% confidence interval; ROR: reporting odds ratio

Adverse event	Category	Occurrence	No occurrence	ROR (97.5% CI)	p-value
Acute kidney injury	Observed value	958	4,577	3.198 (2.808,∞)	<0.001
	Expected value	340	5,195		
Renal failure	Observed value	851	4,684	4.044 (3.484,∞)	<0.001
	Expected value	238	5,297		
Chronic kidney disease	Observed value	1,511	4,024	7.467 (6.512,∞)	<0.001
	Expected value	265	5,270		

To further enhance the versatility of the proposed model, Appendix A includes an R code that reproduces the results presented in Table 8 using the summary result for acute kidney injury from Table 7. This code is designed to output not only the test of ROR results but also the p-value for Fisher's exact test. Researchers can readily apply the proposed model to other studies.

Application 2 results

This study investigated the DDIs between furosemide (drug A) [21] and perindopril (drug B) [22] with respect to nausea, headache, and back pain. Table 9 presents a summary of patient background variables (sex, age, and weight) for each group. Similar to Example 1, the data in Example 2 also exhibited considerable missing information, with 6% to 10% for sex data, 10% to 25% for age data, and 50% to 70% for weight data. Table 9 also provides an overview of the proportions of adverse events by group. For nausea, the observed proportions were q_A_ = 4.7%, q_B_ = 5.0%, and q_AB_ = 3.1%. For headache, the observed proportions were q_A_ = 3.0%, q_B_ = 4.5%, and q_AB_ = 1.8%. For back pain, the observed proportions were q_A_ = 1.7%, q_B_ = 1.9%, and q_AB_ = 1.1%. To select the appropriate scenario, we checked whether q_AB_ < q_B_ was satisfied for these three adverse events. For nausea, q_AB_ = 3.1% < q_B_ = 5.0%. Similarly, for headache, q_AB_ = 1.8% < q_B_ = 4.5%, and for back pain, q_AB_ = 1.1% < q_B_ = 1.9%. As the condition was met for all three adverse events, they were considered potential candidates for negative DDIs. Consequently, the proposed model with c = 1 was employed to conduct two tests, and the results were summarized in Table 10. The ROR with the upper bound of the 97.5% CI and p-value for nausea were 0.608 (97.5%: -∞, 0.715), p < 0.001; for headache, they were 0.389 (97.5% CI: -∞, 0.474), p < 0.001; and for back pain, they were 0.594 (97.5%: -∞, 0.775), p < 0.001. Negative DDIs were confirmed for these three adverse events.

**Table 9:** Summary of patient background variables and adverse events for furosemide (drug A) and perindopril (drug B). Sex is summarized as frequency (proportion), age and weight are summarized as median (Q1–Q3), and missing data for each patient background variable are summarized as frequency (proportion). Q1: first quartile; Q3: third quartile

	Drug A group	Drug B group	Drugs AB group
	N = 340,725	N = 37,085	N = 7,913
Sex			
Female, n (%)	175,684 (51.6)	15,790 (42.6)	3,268 (41.3)
Male, n (%)	142,991 (42.0)	17,902 (48.3)	3,863 (48.8)
Missing, n (%)	22,050 (6.5)	3,393 (9.1)	782 (9.9)
Age (years)			
Median	70.0	67.0	74.0
Q1–Q3	60.0–79.0	58.0–76.0	65.0–82.0
Missing, n (%)	76,268 (22.4)	6,220 (16.8)	1,163 (14.7)
Weight, kg			
Median	78.5	78.0	79.2
Q1–Q3	63.5–96.0	66.0–91.0	65.0–94.0
Missing, n (%)	201,786 (59.2)	23,375 (63.0)	5,092 (64.3)
Adverse event			
Nausea, n (%)	15,876 (4.7)	1,866 (5.0)	247 (3.1)
Headache, n (%)	10,266 (3.0)	1,663 (4.5)	142 (1.8)
Back pain, n (%)	5,858 (1.7)	689 (1.9)	85 (1.1)

**Table 10:** ROR and p-value for negative interaction term in each adverse event with furosemide (drug A) and perindopril (drug B). 97.5% CI: the upper bound of the 97.5% confidence interval; ROR: reporting odds ratio.

Adverse event	Category	Occurrence	No occurrence	ROR (97.5% CI)	p-value
Nausea	Observed value	247	7,666	0.608 (-∞,0.715)	<0.001
	Expected value	398	7,515		
Headache	Observed value	142	7,771	0.389 (-∞,0.474)	<0.001
	Expected value	355	7,558		
Back pain	Observed value	88	7,825	0.594 (-∞,0.775)	<0.001
	Expected value	147	7,766		

Overall, these applications demonstrate the practical utility of the proposed model in identifying both positive and negative DDIs using FAERS data while addressing limitations related to missing patient background information.

## Discussion

The introduction of novel pharmaceuticals demands a comprehensive investigation of potential DDIs with existing medications. However, conducting exhaustive studies on all potential DDIs prior to drug approval is impractical, as demonstrated by the rapid development of COVID-19 therapeutics [23]. Consequently, utilizing large-scale databases for post-approval DDI studies emerges as a viable alternative [24]. These databases frequently suffer from insufficient patient background information that could serve as critical covariates. While sex and age data are typically provided, essential information such as laboratory test values, comorbidities, lifestyle factors, and genetic information is often missing or incomplete [25]. Moreover, even for sex and age, the prevalence of missing data can range from 5% to 20% in large-scale databases [9]. Although data imputation methods exist, achieving high-precision imputation becomes challenging due to limited patient background information [26].

The proposed model's strength lies in its ability to address challenges associated with incomplete patient background data. Simulation studies confirmed the model's robustness, maintaining appropriate Type I error rates across various settings. While prioritizing Type I error control occasionally resulted in low detection power, this was primarily observed in cases with small interaction terms. Importantly, the model effectively distinguishes between statistical and clinical significance [18], potentially mitigating the detection of statistically significant but clinically irrelevant differences. For more substantial interaction terms, the model demonstrated high detection power, aligning statistical significance with clinical relevance. This characteristic enhances the model's practical utility in clinical settings, providing healthcare professionals with a more nuanced tool for identifying meaningful DDIs.

In Applications 1 and 2, positive DDIs were observed between tramadol and ciprofloxacin regarding acute kidney injury, renal failure, and chronic kidney disease. These DDIs can be attributed to the individual nephrotoxic characteristics of both medications, which may be synergistically amplified during concurrent administration [27,28]. Conversely, the investigation of furosemide and perindopril observed negative DDIs related to nausea, headache, and back pain. While the findings provide valuable insights, it is crucial to acknowledge the inherent limitations of database research. Future research should prioritize controlled clinical studies to validate these database-derived observations and elucidate the underlying mechanisms of these DDIs.

The study encountered several limitations. The utilized database potentially provided limited patient background information and contained significant missing data, which could impact the findings' robustness. Reliance on spontaneous adverse event reporting introduces the possibility of reporting bias. Furthermore, databases like the FAERS do not capture reports of non-occurring adverse events, making it impossible to calculate true adverse event incidence.

Given these inherent research limitations, the results should be viewed as a preliminary investigation rather than definitive conclusions. The comprehensive exploration of potential DDIs remains challenging due to the vast number of possible drug combinations. Nevertheless, this study provides a valuable foundation by identifying specific drug combinations that warrant further investigation.

## Conclusions

This study offers valuable insights into potential DDIs and presents a methodological framework for future investigations. By focusing on the significant DDIs identified, subsequent research can more efficiently explore DDIs with meaningful clinical implications, ultimately contributing to improved patient safety and pharmacological practices. The proposed model's ability to handle missing data while maintaining appropriate Type I error rates offers a promising approach for future DDI studies using large-scale databases.

## Appendices

Appendix A

We provide sample R code to reproduce the result presented in Table 8 for acute kidney injury, using the summary result for acute kidney injury from Table 7. By modifying the nA, nB, nAB, xA, xB, and xAB values in the first two lines of the R code, researchers can easily apply the proposed model to analyze other datasets.

nA<-204368; nB<-81423; nAB<-5535 xA<-4685; xB<-3131; xAB<-958 Table_AE<-rbind(paste("N =",c(nA,nB,nAB)), paste0(c(xA,xB,xAB)," (",sprintf("%.1f",c(xA,xB,xAB)/c(nA,nB,nAB)*100),")")) colnames(Table_AE)<-c("Drug A group","Drug B group","Drugs AB group") rownames(Table_AE)<-c("","Adverse event, n(%)") calculate_ddi<-function(xAB,nAB,est_N,alternative){ ROR<-(xAB/(nAB-xAB))/(est_N/(nAB-est_N)) se_log_ROR<-sqrt(1/xAB+1/(nAB-xAB)+1/est_N+1/(nAB-est_N)) if(alternative=="greater"){ ROR_p<-1-pnorm(log(ROR)/se_log_ROR) ci<-exp(log(ROR)-qnorm(0.975)*se_log_ROR) ci_str<-paste0(format(round(ci,3),nsmall=3),", ∞)") }else{ ROR_p<-pnorm(log(ROR)/se_log_ROR) ci<-exp(log(ROR)+qnorm(0.975)*se_log_ROR) ci_str<-paste0("-∞, ",format(round(ci,3),nsmall=3),")") } mat<-matrix(c(xAB,est_N,nAB-xAB,nAB-est_N),2,2) Fisher_p<-fisher.test(mat,alternative=alternative)$p.value ROR_p<-ifelse(ROR_p<0.001,"<0.001",ifelse(ROR_p>0.999,">0.999", format(round(ROR_p,3),nsmall=3))) Fisher_p<-ifelse(Fisher_p<0.001,"<0.001",ifelse(Fisher_p>0.999,">0.999", format(round(Fisher_p,3),nsmall=3))) return(list(ROR=ROR,ci_str=ci_str,ROR_p=ROR_p,Fisher_p=Fisher_p,mat=mat)) } est_p<-xA/nA+xB/nB est_N<-round(nAB*est_p,0) pos_ddi<-calculate_ddi(xAB,nAB,est_N,"greater") est_p<-max(xA/nA,xB/nB) est_N<-round(nAB*est_p,0) neg_ddi<-calculate_ddi(xAB,nAB,est_N,"less") create_table<-function(ddi_result,c_value){ c_t<-cbind(c(c_value,""),c("Observed value","Expected value"),ddi_result$mat, c(paste0(format(round(ddi_result$ROR,3),nsmall=3)," (",ddi_result$ci_str),""), c(ddi_result$ROR_p,""),c(ddi_result$Fisher_p,"") ) colnames(c_t)<-c("c","Category","Occurrence","No occurrence","ROR (97.5% CI)","ROR_p","Fisher_p"); return(c_t) } Table_positiveDDI<-create_table(pos_ddi,0) Table_negativeDDI<-create_table(neg_ddi,1) Table_AE Table_positiveDDI Table_negativeDDI
